# Profiling High-Intensity Message Senders Among Older Adult Patient Portal Users

**DOI:** 10.1093/geroni/igaf122.379

**Published:** 2025-12-31

**Authors:** Aleksandra Wec, Jenni Seale Reiff, Jennifer Wolff

**Affiliations:** Johns Hopkins Bloomberg School of Public Health, Baltimore, Maryland, United States; Johns Hopkins Bloomberg School of Public Health, Baltimore, Maryland, United States; Johns Hopkins University - School of Public Health, Baltimore, Maryland, United States

## Abstract

Rising secure messaging volume in patient portals has led to increasing demand on clinician time. Little is known about patients who send the most messages. We develop a profile of high-intensity secure message senders from a sample of 16,023 older adults who sent 199,952 messages to clinicians at an academic health system over 12 months. High-intensity messaging was defined using outlier analysis of aggregate message volume during the 12-month observation period. Additional measures of high-intensity messaging included identifying concentrated periods of messaging, message length, and ratios of messages to portal logins and to healthcare encounters. We compared socio-demographic characteristics, health status, and messaging behaviors of high-intensity senders to all other message senders. Of the total sample, 1,298 patients (8.1%) were classified as high-intensity message senders; these patients accounted for 39.7% of total messages. High-intensity message senders, compared to all other message senders, were more likely to be white (80.4% vs. 72.5%, p < 0.001), have higher comorbidity scores (2.6 vs. 1.8, p < 0.001), to have diagnosed cancer (35.8% vs. 22.8%, p < 0.001) or dementia (8.3% vs. 6.1%, p < 0.002). They were also more likely to have concentrated messaging periods, send longer messages, and have greater patient portal use and higher healthcare utilization in relation to the number of messages they sent. High-intensity message senders form a small, distinct group of older adults who contribute disproportionately to message volume. Better understanding the profile of high-intensity message senders may inform targeted initiatives seeking to improve patient care while easing clinicians’ inbox workload.

